# Effection of monoplanar pedicle screw on facet joint degeneration in thoracolumbar vertebral fractures

**DOI:** 10.1186/s12891-022-05360-3

**Published:** 2022-04-30

**Authors:** Bin Ye, Zhengxu Ye, Ming Yan, Peipei Huang, Zhipeng Tu, Zhe Wang, Zhuojing Luo, Xueyu Hu

**Affiliations:** Department of Orthopedics, Xijing Hospital, Air Force Medical University of PLA, No. 169 West Changle Road, Shaanxi Province, 710032 Xi’an, China

**Keywords:** Monoplanar pedicle screws, Fixed-axis pedicle screw, Thoracolumbar vertebral fractures, Facet joints, Violation rate

## Abstract

**Background:**

This study aimed to compare the clinical outcomes and effect on instrument-related facet joints between fixed-axis pedicle screw (FAPS) and monoplanar pedicle screw (MPPS).

**Methods:**

816 pedicle screws of 204 patients with thoracolumbar vertebral fractures (TLVF) who underwent internal fixation surgery were analyzed in this retrospective study. All patients were divided into two groups (FAPS and MPPS). Preoperative, immediate postoperative, and 12–18-months postoperative CT and X-ray, and clinical data, including demographics, preoperative and immediate postoperative Visual Analogue Scale (VAS), blood loss (BL), operation time (OT) and hospital stay time (HST), were collected. Facet joint violation and degeneration grade were evaluated by CT according to Babu’s criteria and Weishaupt’s criteria respectively, and preoperative, immediate postoperative and 12–18-months postoperative anterior body compression index (ABCI) were measured by X-ray.

**Results:**

Postoperative VAS of two groups was lower than preoperative VAS (*p* < 0.05). BL, OT, and HST were less in MPPS than FAPS, and the difference was statistically significant in BL and HST (*p* < 0.05) but no in OT (*p* > 0.05). Immediate postoperative and 12–18-months postoperative ABCI were significantly higher than preoperative (p < 0.05), and the difference of ABCI between immediate postoperative and 12–18-months postoperative were not significant in two groups (*p* > 0.05). Total violation rate (VR) was about 1.35% (11/816) and FAPS had a lower VR than MPPS, but no significant (*p* > 0.05). Weishaupt’s criteria revealed that average class (AC) was 0.69 in FAPS and 0.67 in MPPS, and the distribution of degenerated facet joints in two groups did not differ preoperatively (*p* > 0.05). In 12–18 months postoperatively, AC was significantly higher in FAPS than in MPPS, and the distribution of degenerated facet joints in two groups was significantly different (*p* < 0.05). The comparison of cranial to caudal joints in two groups revealed that cranial joints had more severe degeneration than caudal joints.

**Conclusions:**

The findings suggested that both MPPS and FAPS were effective for patients with TLVF, but MPPS by percutaneous may be a better choice to avoid adjacent segment degeneration, especially the surgery-involved facet joints degeneration.

## Background

Posterior pedicle screw fixation has stronger fixation strength than other instrumentations and has been widely used in spinal diseases since first introduced by Boucher in 1959 [1–3]. The typical example is unstable thoracolumbar vertebral fractures (TLVF), mainly caused by trauma, could obtain tough internal fixation to correct the deformity and maintain sagittal balance by posterior pedicle screw insertion.

To date, for TLVF patients [4, 5], fixed-axis pedicle screw (FAPS) design was used as the instrumentation in internal fixation surgery by open technique as it is difficult to insert the rod via a minimally invasive technique. With the development of damage control surgery in Orthopedics, surgeons attempted to fix the spine fracture by a minimally invasive technique and simplify the process of rod insertion. Thus, polyaxial pedicle screw (PAPS) was used for TLVF, which was widely acceptable. The new screw tail design could swivel freely in any plane, solve the difficulty, and facilitate coupling of the screw with a longitudinal rod; however, the stiffness in the sagittal plane decreased [6–9]. In order to combine two advantages of strong fixation strength and facilitating rod insertion, we made a novel screw tail design, termed monoplanar pedicle screws (MPPS), which is mobile in the axial plane to facilitate rod insertion but fixed in the sagittal plane to maintain stability during flexion loads and increase the stiffness of instrumentation in flexion and extension, at least theoretically. The present study aimed to investigate the mechanical properties of MPPS that might contribute to the sagittal balance and reduce the risk of correction loss [10].

With the emergence of minimally invasive techniques, percutaneous pedicle screw (PPS) has gained increasing attention. Moreover, PPS has the advantages of avoiding extensive detachment of muscle, thereby reducing blood loss (BL), alleviating postoperative pain, and accelerating recovery and rehabilitation. However, the learning curve of PPS is steeper than that of open techniques. The operation under indirect vision increases the difficulty of placing screws and the incidence of instrumentation-related facet joint violation. Several studies reported that the rates of instrumentation-related facet joint violation ranged from 7 to 58% using open technique and from 11 to 100% with PPS placement technique [11–13], which was positively correlated with the degenerative changes of facet joints [13, 14].

In terms of the mobile situation of screw tail, pedicle screws could be easily classified into three types, FAPS, PAPS, and MPPS. Different designs had varied biomechanical properties, advantages, and disadvantages, which in turn affects the relevant facet joints, especially the adjacent segments. All types of pedicle screws, except MPPS, have been verified for their influence on adjacent segments. Thus, the present study aimed to compare the influence of FAPS and MPPS on the operative and adjacent segment facet joint degenerative changes.

## Methods

### Patient cohort

We reviewed our clinical database and identified 235 patients with TLVF who underwent a posterior fracture internal fixation with FAPS between July 2010 and May 2015, or MPPS between June 2015 and June 2019 in Xijing Hospital, Xi’an, Shaanxi Province, China.

#### Inclusion Criteria

Patients with one level spinal fracture in a thoracolumbar vertebra (T12–L2), type A fracture according to AO Spine Thoracolumbar Spine Injury Classification System [15], bilateral pedicle screw fixation in the levels of superior and inferior of fracture body without decompression and fusion, and follow-up data including X-ray and CT scan preoperatively, immediate postoperatively, and 12–18-months postoperatively.

#### Exclusion Criteria

**Fig. 1:**
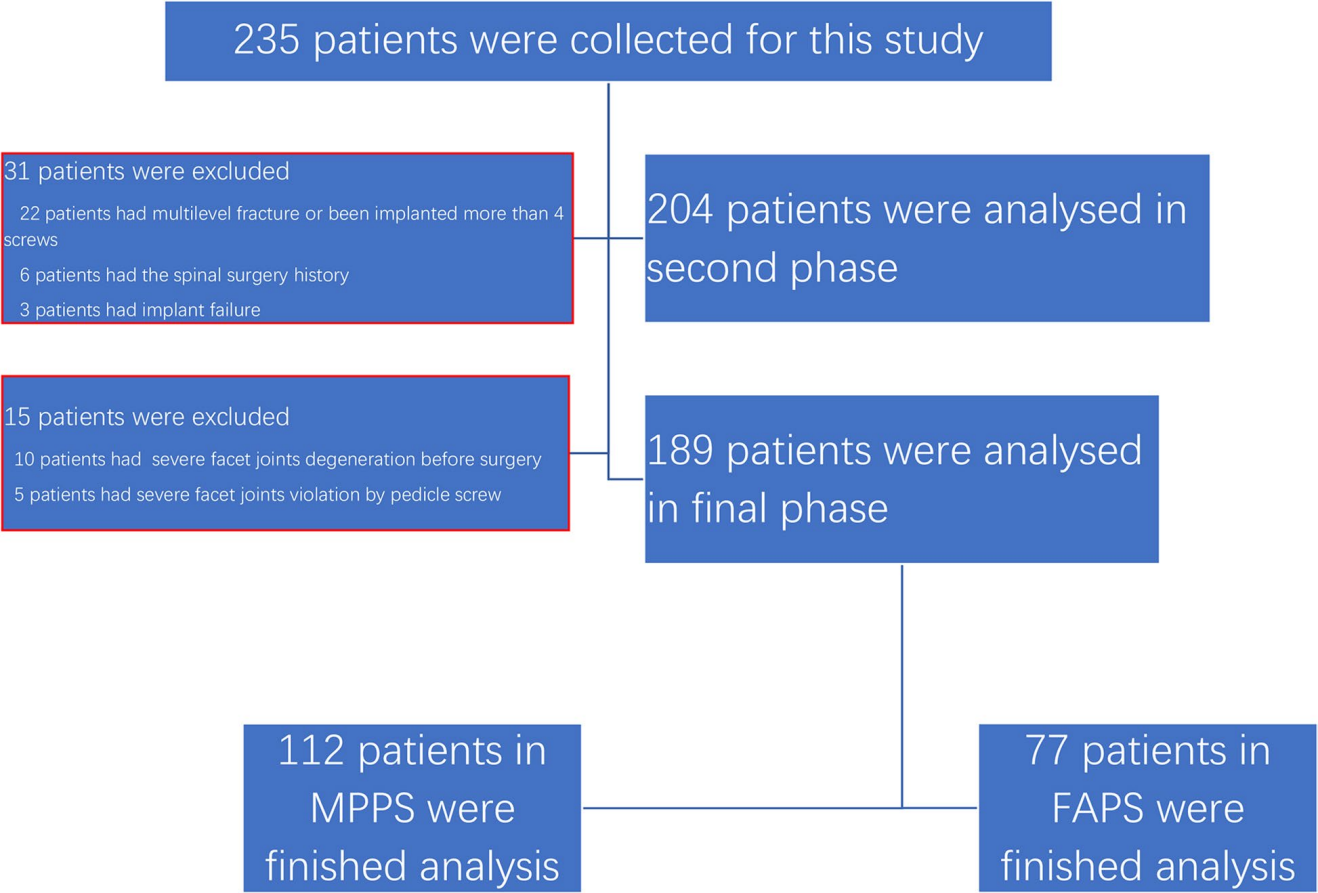
Consolidated Standards of Reporting Trials (CONSORT) diagram for the study

Previous spinal surgery, patients with spinal diseases, multilevel fracture or severe degeneration (grade 2–3) in involved facet joints before surgery, implant failure in the follow-up period, fracture involving the evaluated facet joints or pedicle screw violated the involved facet joints severely (grade 2–3). 189/235 patients fulfilled the inclusion criteria and were divided into two groups (FAPS group and MPPS group) according to the types of instrumentation. The Consolidated Standards of Reporting Trials for this study is illustrated in Fig. 1.

### Surgical technique

The pedicle screw was placed by open approach in the FAPS group but the percutaneous approach in the MPPS group. All operations were completed by a senior surgeon, and all involved patients were positioned prone on a radiolucent operating table under general anesthesia, and conventional C-arm fluoroscopy was utilized for the entire procedure.

#### Open approach

After a midline surgical exposure, the soft tissues were dissected to the base portion of the transverse processes. The anatomical hallmarks of the superior and inferior margins of the transverse process, the lateral border of the pars, and the lateral margin of the facet were used to find the insertion hole for the pedicle screw. After the entry point was identified, the trajectory was projected according to the preoperative CT data. Then, the locating pin was inserted into the prepared trajectory and confirmed that it was in the appropriate position by anteroposterior and lateral projections. Then, the satisfactory path was found, the screw hole was tapped, and the four walls of trajectory were be explored to be intact using the ball-tipped probe. The appropriately sized pedicle screw was inserted into the prepared hole. Then, a titanium rod was assembled into a pre-shape to check the correction of vertebral height and sagittal alignment and locked into the screw tail with nuts. When necessary, appropriate distraction could be carried out to recover the vertebral height and sagittal alignment.

#### Percutaneous approach

Firstly, the surface location of the pedicle was marked by the anteroposterior projection. Then, the skin and fascia were incised. A parallel incision with the spinous process was made in the superior vertebra to facilitate rod insertion but perpendicular incision to spinous process in the inferior vertebra for easing screw abduction. The puncture needle was inserted into the pedicle intramuscularly. The entry point of the needle was the lateral end at the slightly cranial margin of the pedicle, and the trajectory was projected according to the preoperative CT data. In order to reduce the puncture-related effect on facet joint, we used the method of continuous fluoroscopy to ensure the appropriate entry point. After confirming the appropriate position by anteroposterior and lateral projections, the inner needle was pulled out, the guidewire was inserted, and then the outer needle was pulled out. After the satisfactory path was identified, the screw hole was tapped along the wire with screw taps. The appropriately-sized pedicle screw was inserted into the prepared screw hole, wherein a titanium rod was inserted and pre-shaped to check the correction of the vertebral height and sagittal alignment. Finally, the rod was assembled into the screw tail and locked with nuts. When necessary, appropriate distraction was carried out to recover the vertebral height and sagittal alignment.

### Clinical data evaluation

The patients’ Visual Analogue Scale (VAS) preoperatively and postoperatively was compared to evaluate the efficiency of the operation. The surgical parameters, including BL, operation time (OT) and hospital stay time (HST), were used to evaluate the surgical damage of the two types of surgical approach.

### Radiographic evaluation

**Fig. 2:**
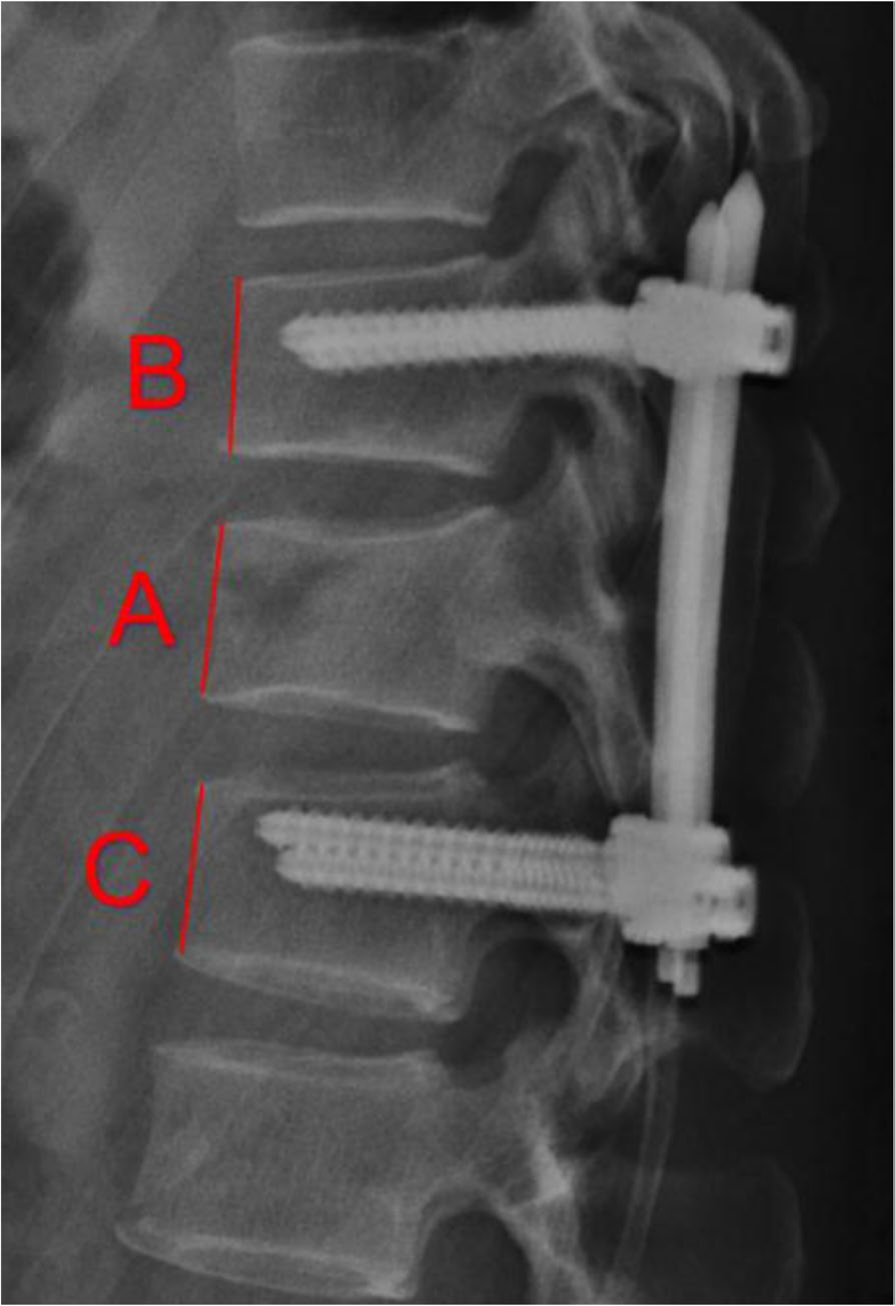
Measurement method of anterior body compression index (ABCI). A, B and C represent the anterior height of each vertebral. ABCI = 2A/(B + C)

**Fig. 3:**
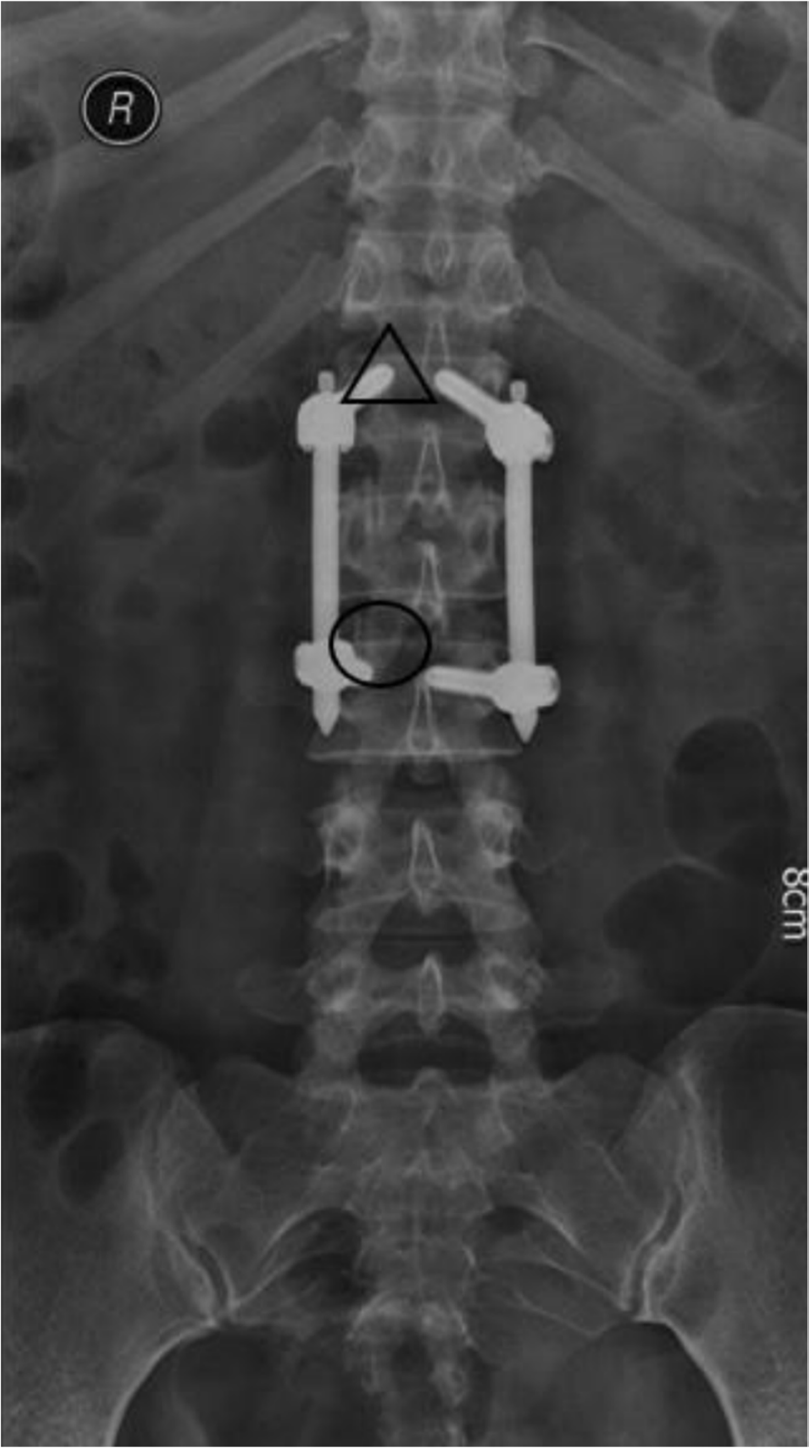
X-ray view of the cranial and caudal facet joints for each patient in the study. ○ shows the caudal facet joints, and △ shows the cranial facet joints

All preoperative, immediate postoperative, and 12–18 months postoperative thin-cut CT scans and X-ray had been obtained at our institute. Preoperative, immediate postoperative, and 12–18 months postoperative anterior body compression index (ABCI), measured by X-ray, was applied to reflect the restoration of the fractured vertebra body and evaluate the correction loss during follow-up (Fig. 2). Preoperative and 12–18-months postoperative CT data were used to classify the degenerative changes of the involving facet joints, and immediate postoperative CT was used to evaluate the facet joint violation by pedicle screw. In this study, the involved facet joints included superior and inferior facet joints (Fig. 3). The superior facet joints were defined as the superior facet of the cranial transfixed vertebra and the inferior facet of the cranial contiguous vertebra. The inferior one was represented by the superior facet of the distal transfixed vertebra and the inferior facet of the fractured vertebra.

**Fig. 4:**
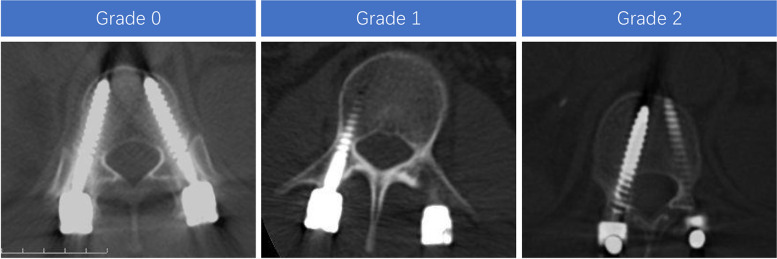
Representative examples of CT scans demonstrating the different grades of facet joints violation

**Fig. 5:**
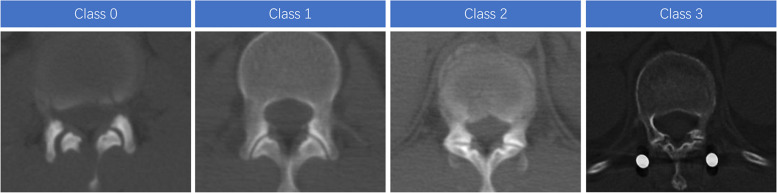
Representative examples of CT scans demonstrating the different classes of facet joints degeneration

The criteria for defining a facet joint violation and degenerative change were established before the start of the study. Babu’s criteria were used to evaluate the impingement of the facet joints with screws (Table 1, Fig. 4) [16]. Weishaupt’s criteria were used to evaluate the facet joint degeneration (Table 2, Fig. 5) [17]. The data of the consecutive patients were analyzed and graded by two surgeons. The final decision was made by the third surgeon in case of discrepancies.

**Table 1:** Babu’s criteria for Facet joints violation grade

Grade 0	Screw not in facet
Grade 1	Screw in lateral facet but not in facet articulation
Grade 2	Penetration of facet articulation by screw
Grade 3	Screw travels within facet articulation

**Table 2:** Weishaupt’s criteria for Facet joint degenerative class

Class 0	Normal facet joint space (2 ~ 4 mm width)
Class 1	Narrowing of the facet joint space (< 2 mm) and / or small osteophytes and / or mild hypertrophy of the articular process
Class 2	Narrowing of the facet joint space and / or moderate osteophytes and / or moderate hypertrophy of the articular process and / or mild subarticular bone erosions
Class 3	Narrowing of the facet joint space and / or large osteophytes and / or severe hypertrophy of the articular process and / or severe subarticular bone erosions and / or subchondral cysts

### Statistical analysis

The measurement data were expressed as mean ± standard deviation. All statistical analyses were performed using SPSS statistics version 25. The differences in measurement data were analyzed using a Student’s t-test, enumeration data using chi-square, and ranked data using rank-sum test to determine significant differences. For all tests, *p* < 0.05 was considered statistically significant.

## Results

### Study population

**Fig. 6:**
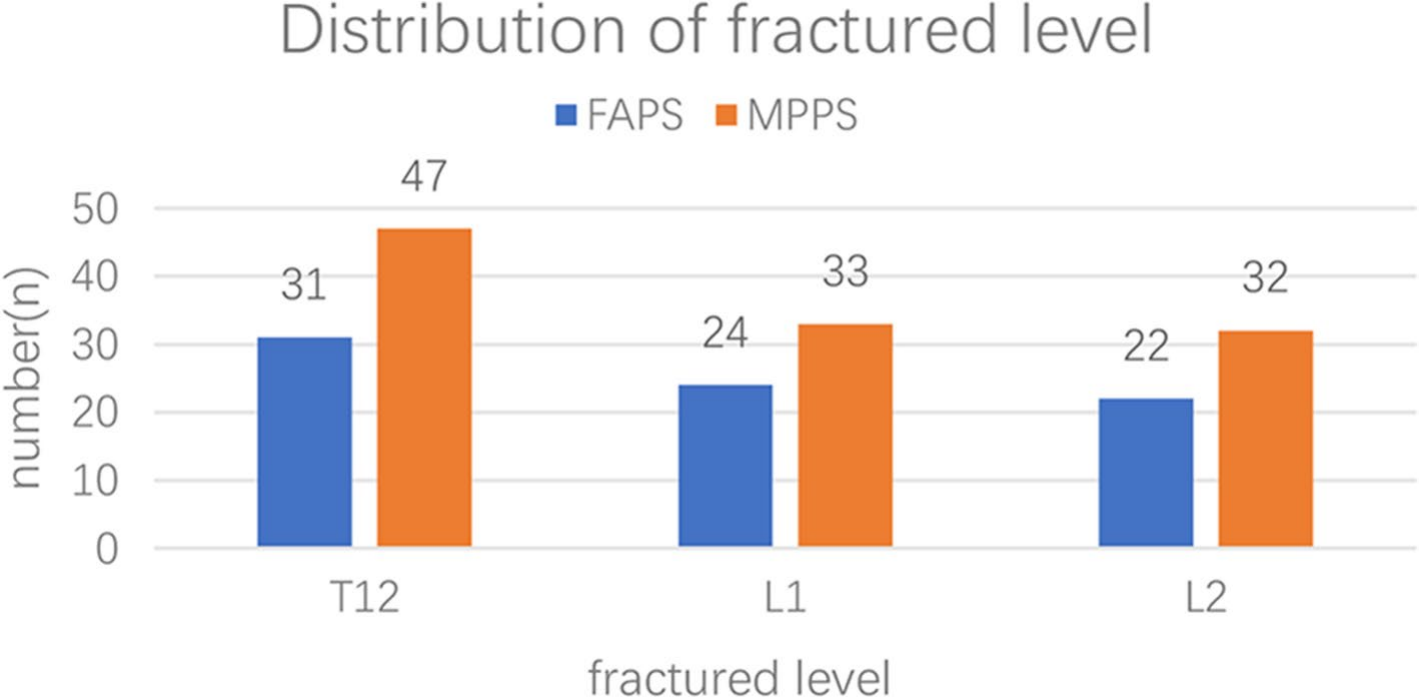
Distribution of fractured level

Table 3 and Fig. 6 summarized the baseline characteristics of patients who underwent surgery for posterior fracture internal fixation with FAPS or MPPS. The average age of the 77 patients (male:female = 36:41) in the FAPS group was 39.6 ± 12.2 years, and fractured level occurred at T12-L2 (T12:L1:L2 = 31:24:22). The average age of the 112 patients (male:female = 58:54) in the MPPS group was 43.7 ± 18.3 years, and the fractural level occurred at T12-L2 (T12:L1:L2 = 47:33:32), However, no statistically significant difference was detected in the age or distribution of gender and fractured level between the two groups (*p* > 0.05).

**Table 3:** Demographic characteristics and damage distribution of the patients

Parameter		FAPS group	MPPS group	P Value
No. of patients		77	112	/
Gender distribution	male	36	58	= 0.5^a^
	female	41	54	
Age (mean ± SD)		39.6 ± 12.2	43.7 ± 18.3	> 0.05^b^
Fractured level	T12	31	47	> 0.5^a^
	L1	24	33	
	L2	22	32	

^a^The values were given by Chi-square;

^b^The value was given by Student's t test

### Clinical data analysis

The preoperative VAS (8.3 ± 1.1) in the FAPS group was obviously higher than that postoperatively (2.3 ± 0.6) (*p* < 0.05). Similarly, a significant decrease was noted in MPPS between preoperation (8.0 ± 1.4) and postoperation (2.1 ± 1.0) (*p* < 0.05). Moreover, BL, OT, and HST were less in MPPS than FAPS, and the difference was statistically significant in BL and HST (*p* < 0.05) but no significant in OT (*p* > 0.05) (Table 4).

**Table 4:** Clinical date related to the operation approach

Parameter		FAPS (*n* = 77)	MPPS (*n* = 112)	*P* Value
VAS (mean ± SD)	Pre-operation	8.3 ± 1.1	8.0 ± 1.4	/
	Post-operation	2.3 ± 0.6	2.1 ± 1.0	/
*P* Value		< 0.001	< 0.001	/
BL (ml, mean ± SD)		216.4 ± 77.9	55.7 ± 43.3	< 0.001
OT (min, mean ± SD)		93.1 ± 20.3	89.6 ± 25.4	> 0.2
HST (day, mean ± SD)		6.85 ± 2.3	4.71 ± 1.5	< 0.001

BL: Blooding Loss; OT: Operation Time; HST: Hospital Stay Time;

All values were given by Student's t test

### Radiographic data analysis

In the primary 235 patients, 31 patients were excluded for multilevel fracture, spinal surgery history or implant failure.7

In the second phase, 10 patients were excluded for severe joints degeneration, and 11 facet joints of 5 patients (3 joints of 1 patient in FAPS, 8 joints of 4 patients in MPPS) were excluded for severe joints violation by pedicle screw. According to Babu’s criteria, the violation rate (VR = (no. of Grade 2 + no. of Grade 3) / (no. of implanted screws)) was about 1.35%. In the FAPS group, the VR was 0.91%, which was lower than 1.64% in the MPPS group, albeit the difference was not statistically significant (*p* > 0.05) (Table 5).

**Table 5:** Summary of facet joints violation by Babu’s grading criteria

Babu’s criteria	FAPS (*n* = 328)	MPPS (*n* = 488)	Total (*n* = 816)	*P* Value
Grade 0	303	431	734	0.058
Grade 1	22	49	71	
Grade 2	3	8	11	
Grade 3	0	0	0	
VR	0.91%	1.64%	1.35%	/

VR (Violation Rate) = (no. of Grade 2 + no. of Grade 3) / (no. of implanted screws);

The value was given by Rank Sum test;

15 patients with severe facet joints degeneration before surgery or severe facet joints violation by pedicle screw were analysed at the table

189 patients were analysed in final phase. According to Weishaupt’s criteria, 308 joints of 77 patients were assessed preoperatively in the FAPS group, and no. of Class 0–Class 1 were 96–212, respectively. 448 joints of 112 patients assessed in the MPPS group, and no. of Class 0–Class 1 were 147–301, respectively. The distribution of degenerated facet joints in the two groups was similar before surgery, and the average class (AC) was 0.69 in the FAPS group and 0.67 in the MPPS group (*p* > 0.05). However, a significant difference was noted in 12–18 months postoperatively between the two groups, and the AC was higher in the FAPS group (AC = 1.85, △AC = 1.16) than in the MPPS group (AC = 1.18, △AC = 0.51) (*p* < 0.05). Moreover, we found that facet joints had more severe degeneration at the cranial level [△AC = 1.49 (2.16–0.67) in the FAPS group, △AC = 0.66 (1.32–0.66) in the MPPS group] than at the caudal level [△AC = 0.83 (1.54–0.71) in the FAPS group, △AC = 0.35 (1.04–0.69) in the MPPS group] after surgery (Table 6). As the Table 7 shows that ABCI in two groups increased significantly immediately after fixation operation, and the difference were significant (*p* < 0.05). Until 12–18 months post-operation, ABCI were maintained well (*p* > 0.05).

**Table 6:** Summary of all involved facet joints degeneration class by Weishaupt’s criteria

	Pathria’s criteria	FAPS			MPPS			*P* Value
		n _(cranial)_ = 154	n _(caudal)_ = 154	n _(FAPS)_ = 308	n _(cranial)_ = 224	n _(caudal)_ = 224	n _(MPPS)_ = 448	
Pre-op	Class 0	51	45	96	77	70	147	0.635
	Class 1	103	109	212	147	154	301	
	AC	0.67	0.71	0.69	0.66	0.69	0.67	/
12–18 months post-op	Class 0	7	12	19	39	43	82	0.000
	Class 1	18	71	89	101	142	243	
	Class 2	73	47	120	57	25	82	
	Class 3	56	24	80	27	14	41	
	AC	2.16	1.54	1.85	1.32	1.04	1.18	/
△AC		1.49	0.83	1.16	0.66	0.35	0.51	/

AC: Average Class; △AC = post-op AC—pre-op AC;

All values were given by Rank Sum test

**Table 7:** Summary of ABCI measured by X-ray

		FAPS group	P Value	MPPS group	*P* Value
ABCI (%)	Pre-op	57.1 ± 11.2	< 0.001^a^	53.3 ± 8.7	< 0.001^c^
	Immediate post-op	89.2 ± 10.3	/	88.1 ± 9.1	/
	12–18 months post-op	89.0 ± 11.0	> 0.5^b^	86.3 ± 8.9	> 0.1^d^

*ABCI* anterior body compression index, *FAPS* fixed-axis pedicle screw, *MPPS* monoplanar pedicle screw

^a^Represents a significant difference from immediate post-op in FAPS group(*P* < 0.001)

^b^Represents no significant difference from immediate post-op in FAPS group (*P* > 0.5)

^c^Represents a significant difference from immediate post-op in MPPS group (*P* < 0.001)

^d^Represents no significant difference from immediate post-op in MPPS group (*P* > 0.1)

## Discussion

Recently, minimally invasive spinal surgical techniques (MISST) have been the focus of the surgeons due to large potential advantages. The PPS placement technique plays a critical role in MISST, which could reduce the surgical trauma to the surrounding musculature, decrease the BL, and shorten the recovery time. Currently, there are two types of screws (PAPS and MPPS) that could be used in percutaneous surgery. In addition to the advantage of facilitating coupling of the screw with a longitudinal rod, MPPS strengthens the stiffness in the sagittal plane, which combines the advantages of PAPS and FAPS. Firstly, in the current study, two types of pedicle screws implanted by the percutaneous or open approach could be effective measures to decrease VAS and therapy TLVF. Secondly, this study did not show any significantly different OT but distinctly different BL and HST in the two groups, which was consistent with the previous findings [18–22].

Nonetheless, several studies demonstrated a significant facet joint violation in the PPS surgery compared to the open approach [16, 23], which was associated with the degradation of facet joint and adjacent segment. However, the screw insertion accuracy or facet joints violation is related to “surgeon’s learning curve”. Moshirfar et al. reported that 24% of cases exhibited facet joint violation by an open approach [12], and Shah et al. demonstrated 35% facet joint violation by open approach [13]. On the other hand, some studies about PPS placement showed that VR differed from 11.5–50% [24, 25]. Thus, whether the surgical approach resulted in facet joint violation is yet unclear. As a novel design pedicle screw to facet joints, the VR of MPPS has not been reported previously. The present study compared the VR and facet joint degeneration between FAPS and MPPS and found that the FAPS group had lower VR (0.91%) compared to the MPPS group (1.64%) but the difference was no significant (*p* > 0.05). In all the violated facet joints of the two groups, the maximal violation was Grade 1 (71/82), and the remaining was Grade 2 (11/82). No screw violated the facet joints with Grade 3, which benefitted from the use of C-arm because Grade 3 violation usually occurs with pedicle violation. When pedicle violation was detected in surgery, the operator would adjust and replace the screw to avoid stimulating the spinal cord or nerves. To some extent, adjustment would bias the results. The current results revealed that pedicle screw placement by percutaneous approach had a higher rate to violate the facet joints than by open approach because it did not allow gross visualization and tactile feel. On the other hand, we used the method of continuous fluoroscopy to ensure the appropriate entry point in PPS surgery, in order to reduce the puncture-related effect on facet joint, which result in no significant difference of VR between two groups.

The comparison of the degenerative changes between two groups, we excluded the 15 patients with severe facet joints degeneration before surgery or severe facet joints violation by pedicle screw, revealed that the FAPS group had more severe degeneration (△AC = 1.16) than the MPPS group (△AC = 0.51) after surgery, which contradicted the previous cognition that PPS operation with higher facet joint violation rate, which was positively correlated to degenerative changes in facet joints. This phenomenon could be explained by the observation that all severe violated joints (grade 2–3) in this study, which were excluded in the evaluation of joints degeneration, degenerated in various degrees, which was consistent with the literature. However, mildly (grade 1) violated joint was not affected the joint space and caused the joint degeneration, and the larger surgical damage by open approach and concentration of stress due to the biomechanical characteristics of FAPS was ubiquitous, giving rise to the above situation.

Based on the surgical effect on the joints between cranial and caudal level, we concluded that pedicle screw placement accelerated the degenerative process and had more influence on the cranial facet joints than caudal joints. This phenomenon could be attributed to the stress due to fixation across the joints in the cranial joints but shielding of stress in the caudal joints, which accelerated cranial joint degeneration but decelerated caudal joint degeneration.

To the best of our knowledge, this is the first study to examine both the accuracy and rate of facet joint violation for MPPS. Facet joints violation by pedicle screw accelerates the degeneration but also weakens the pullout strength [26]. Subsequently, we concluded that implanting the pedicle screw by the open approach has lower VR than by the percutaneous approach. However, several factors affect the accuracy, including device resolution, operator’s experience, and method of screw placement. Chen et al. focused on the starting point of pedicle screw placement. The study focused on the incidence of violation, which was 100%, as assessed by the Roy-Camille method but 25% using the Weinstein method [27]. The insertion of MPPS is similar to PAPS by percutaneous approach. On the other hand, the FAPS was placed by open approach. In this study, the attending surgeon has more than 20 years work experience, and finished more than 5,000 open approach pedicle screw placements and 3,000 minimally invasive pedicle screw placement by percutaneous. So that the placement of screws, no matter by percutaneous or open approach, have been very familiar to him. Otherwise, the results of this study showed that the whole VR was extremely low, about 1.35%, 0.91% in FAPS group and 1.64% in MPPS group. Considering the above reasons, the effect of learning curve on the results was negligible. Nevertheless, the present study has several limitations that require further investigation. Firstly, the sample size is not sufficiently large. Secondly, the follow-up duration is short. When the instrumentations were removed 12–18 months postoperatively, continuous data collection was challenging. In addition, a prospective study to analyze the risk factors for violation caused by pedicle screws was not easy to conduct due to the low incidence of the condition. Lastly, in this retrospective study, all operations were done by one senior surgeon, which could avoid the bias between different practitioners but may arise new biases because of the surgeon's habits and practices. In following study, we will try to do a prospective multicenter study design to compare the difference between FAPS and MPPS, in order to explain the difference more scientifically.

## Conclusions

This study compared the clinical outcomes and the side effects on the facet joints between MPPS and FAPS, evaluating the placement of 816 pedicle screws in 204 patients with TLVF who underwent percutaneous or open surgery. According to the results obtained, both MPPS and FAPS are appropriate as effective treatments for patients with TLVF, but MPPS placed by percutaneous approach may be a better choice to avoid adjacent segment degeneration, especially the surgery-involved facet joint degeneration.

## Data Availability

The datasets used and/or analysed during the current study are available from the corresponding author on reasonable request.

## Abbreviations

FAPS: Fixed-axis pedicle screw
MPPS: Monoplanar pedicle screw
TLVF: Thoracolumbar vertebral fractures
VAS: Visual Analogue Scale
BL: Blood loss
OT: Operation time
HST: Hospital stay time
ABCI: Anterior body compression index
VR: Violation rate
AC: Average class
PAPS: Polyaxial pedicle screw
PPS: Percutaneous pedicle screw
MISST: Minimally invasive spinal surgical techniques
